# Full noncontact laser ultrasound: first human data

**DOI:** 10.1038/s41377-019-0229-8

**Published:** 2019-12-20

**Authors:** Xiang Zhang, Jonathan R. Fincke, Charles M. Wynn, Matt R. Johnson, Robert W. Haupt, Brian W. Anthony

**Affiliations:** 1 Department of Mechanical Engineering, Massachusetts Institute of Technology, 77 Massachusetts Ave., Cambridge, MA 02139 USA; 2 Institute for Medical Engineering & Science, Massachusetts Institute of Technology, 45 Carleton St., Cambridge, MA 02142 USA; 3 Lincoln Laboratory, Massachusetts Institute of Technology, 244 Wood Street, Lexington, MA 02421 USA

**Keywords:** Photoacoustics, Biophotonics, Imaging and sensing, Applied optics

## Abstract

Full noncontact laser ultrasound (LUS) imaging has several distinct advantages over current medical ultrasound (US) technologies: elimination of the coupling mediums (gel/water), operator-independent image quality, improved repeatability, and volumetric imaging. Current light-based ultrasound utilizing tissue-penetrating photoacoustics (PA) generally uses traditional piezoelectric transducers in contact with the imaged tissue or carries an optical fiber detector close to the imaging site. Unlike PA, the LUS design presented here minimizes the optical penetration and specifically restricts optical-to-acoustic energy transduction at the tissue surface, maximizing the generated acoustic source amplitude. With an appropriate optical design and interferometry, any exposed tissue surfaces can become viable acoustic sources and detectors. LUS operates analogously to conventional ultrasound but uses light instead of piezoelectric elements. Here, we present full noncontact LUS results, imaging targets at ~5 cm depths and at a meter-scale standoff from the target surface. Experimental results demonstrating volumetric imaging and the first LUS images on humans are presented, all at eye- and skin-safe optical exposure levels. The progression of LUS imaging from tissue-mimicking phantoms, to excised animal tissue, to humans in vivo is shown, with validation from conventional ultrasound images. The LUS system design insights and results presented here inspire further LUS development and are a significant step toward the clinical implementation of LUS.

## Bioimaging: Tuned-in lasers help solve ultrasound’s touchy issues

A technology that delivers ultrasound images of living tissue without directly touching the target has undergone successful human testing for the first time. The need to contact a patient’s skin with conventional sound-sensitive piezoelectric devices can cause errors in ultrasound imaging when operators apply different amounts of pressure. Xiang Zhang and Brian Anthony from the Massachusetts Institute of Technology, in Cambridge, United States, and colleagues have designed a system that overcomes these limitations by using lasers to create and listen to ultrasound. The new method uses pulsed light to excite a thin upper portion of skin, producing acoustic waves that penetrates deeply into tissue. The ultrasound that scatters back to the surface is then detected with a laser interferometer. Noncontact imaging of 3D objects, animal tissue, and human volunteers detected features comparable to conventional ultrasound.

## Introduction

Modern ultrasonography is well established in diagnostic and interventional medical imaging and is the most commonly used imaging modality for soft tissue^1^. Ultrasound has advantages compared to other imaging methods, including being nonionizing, relatively low cost, and portable. Current embodiments of ultrasound technology range from cart-based bedside systems to portable hand-held devices^1^. Conventional ultrasound imaging requires the placement of piezoelectric transducers in contact with the patient to transmit and then detect reflected and scattered acoustic waves at the body surface. Compared to other imaging modalities, patient contact is a source of variability unique to ultrasound. The imaging clinician applies variable contact forces on the ultrasound probe to the tissue, and the resultant tissue compression causes contact-sensitive images; quantitative ultrasound imaging methods such as shear wave elastography have been shown to be directly sensitive to tissue compression^2–4^. Other contact-sensitive applications, such as remote patient/neonatal monitoring, tracking of wound healing, and imaging of sensitive skin areas, could significantly benefit from a noncontact ultrasound system.

In addition, the freehand reference frame and the cross-sectional or slice nature of conventional ultrasound imaging generates orientation-sensitive images. These two sources of image variability generally complicate longitudinal tracking (monitoring over time) of the tissue morphology using ultrasound. In comparison, MRI and CT have gantry fixed reference frames and generate volumetric images without patient contact. However, frequent imaging for continuous patient monitoring using MRI or CT is prohibitively expensive or would expose patients to significant ionizing radiation in the case of CT.

A volumetric noncontact ultrasound imaging method could resolve many existing limitations and extend ultrasound imaging to broader applications. As presented in this article, full noncontact laser ultrasound (LUS) – employing skin surface photoacoustic sources in combination with laser interferometric detection – generates image features in human studies comparable to conventional ultrasound and could address conventional ultrasound limitations. A fully optical noncontact LUS system is broadly applicable to contact-sensitive imaging applications – elastography, musculoskeletal disease tracking, and imaging of sensitive/painful tissue regions. In addition, LUS may see high usage in critical care and surgical situations where large-area and high-temporal-resolution imaging is often necessary but forgone due to cost, radiation, or inability to safely relocate patients into MRI scanners.

Other approaches making advances toward noncontact and volumetric ultrasound include ultrasound tomography (UST) and photoacoustics (PA) imaging. UST methods typically surround an imaging target with ultrasonic elements within a water tank^5–10^. UST systems can produce volumetric ultrasound images of human tissue comparable to those of MRI or CT^5,11^. However, the geometric constraints of water tanks and the inflexibility of large ultrasonic arrays limit UST systems to highly specific applications, such as breast imaging. Unlike UST, PA approaches – utilizing the conversion of optical energy to acoustic energy via thermoelastic expansion of the tissue – offers a pathway toward compression and coupling-agent-free ultrasound imaging^12,13^. Since the first report of the PA effect over a century ago, modern PA systems are multiwavelength, multipoint, and multicontrast and have been utilized to image length scales spanning microns to centimeters^12–21^. Generally, PA uses an optical source for excitation and traditional piezoelectric elements for detection. Pulsed lasers irradiating biological tissue generate acoustic impulses via optical absorption and induce thermoelastic stress and relaxation within the tissue^22,23^. By tuning the optical wavelength, varying photosensitive absorbers in biological tissue can be selectively imaged^20^. To increase image quality or imaging depth, optical contrast agents such as nanoparticles or dyes can also be injected^18,21,24^. Since PA signal generation is target specific, the spatial location of the optical to acoustic conversion point within the tissue can be localized only *a posteriori*. Depending on the irradiating optical beam, time inversion of multiple recorded acoustic signals may be necessary to localize the source position for PA image reconstruction^25,26^. The PA imaging depth and resolution are object and application specific – dictated by the interaction of the specific light source and the tissue of interest; thus, optical parameters such as wavelength, power, geometric focus, and repetition rate are critical in PA system design^22^.

In contrast to most PA systems, LUS uses an optical detector and is fully noncontact. The optical detection of ultrasound offers increased sensitivity, broader bandwidth, more compact packaging, and true noncontact measurements^27–36^. The LUS technique is broadly used in nondestructive testing (NDT) for remote thickness measurement, fault detection, and material characterization^27,36–38^. More recently, LUS has been demonstrated on tissue-mimicking phantoms, excised tissue samples, and a chicken chorioallantoic membrane^39–44^. However, a full optical ultrasound system for in vivo human imaging has not been previously demonstrated.

In this article, we report the design and evaluation of an eye- and skin-safe, full noncontact, all-optical LUS imaging system evaluated on humans in vivo. Image results on tissue-mimicking phantoms and ex vivo animal tissue are also presented. Unlike an optical source in a PA system – maximizing optical penetration into the tissue, the optical source for the reported LUS system minimizes tissue penetration, specifically to convert optical energy to acoustic energy at the tissue surface. Typical PA systems rely on optical windows, where tissue optical absorption is low, to penetrate deeper into tissue and selectively image optical absorbers such as hemoglobin or other injected optical contrast agents^15^. Since optical attenuation is two to three orders of magnitude higher than acoustic attenuation in tissue, restricting optical conversion to the tissue surface and interrogating with acoustic propagation is significantly more efficient than optical propagation for deep anatomical imaging. By selecting optical wavelengths with a high optical absorption coefficient, optical penetration is minimized, while heat generation for thermoacoustic conversion is maximized. Furthermore, the spatial location of the generated acoustic source can be localized *a priori*, removing the need for array detection and inversion algorithms for source localization. In combination with an optical detector, the LUS system is noncontact and analogous to conventional probe-based ultrasound imaging except that it uses light. Free-space positioning of the optical source and detector points enables volumetric LUS imaging without costly 2D piezoelectric arrays. Imaging results from the LUS system present the first instance of a laser-based ultrasound system tested on human subjects. Results demonstrating LUS system feasibility and system design insights are reported. The human LUS images are encouraging and will motivate further research toward the clinical implementation of noncontact LUS imaging technology.

## Results

### Volumetric 3D Phantom Imaging

The LUS system was initially evaluated on tissue-mimicking phantoms and ex vivo porcine tissue prior to experimentation on human volunteers. A 1540 nm pulsed source laser delivers the optical pulses to excite acoustic waves on the tissue surface, and a 1550 nm continuous wave (CW) Mach-Zehnder Laser Doppler Vibrometer (LDV) measures returning acoustic vibrations on the tissue surface. Both lasers are manufacturer-rated to be eye and skin safe. The source laser and LDV were measured to have 2.3 mJ per pulse and 9.8 mW, respectively. The source laser has a 2 mm beam diameter on the subject surface, and the LDV spot was manually focused to maximize optical backscatter. The reported noise equivalent power for the LDV is <0.5 µm/s/√Hz. We specifically selected wavelengths near 1500 nm to limit optical penetration to the tissue surface and maximize the converted source amplitude. Wavelengths near 1500 nm can leverage the high optical absorption of tissue near 1500 nm for efficient source conversion while maintaining eye and skin safety by having the highest maximum permissible exposure (MPE) limits, namely, 1 J/cm^2^ and 0.1 W/cm^2^ for 1500–1800 nm pulsed and CW lasers, respectively^45,46^. With the safety limits computed using the limiting aperture diameters from the ANSI standard, the corresponding source and detector irradiances are found to be 0.024 J/cm^2^ and 0.1 W/cm^2^, both within the eye and skin MPE limits. No surface enhancements were used for any LUS imaging experiments.

**Fig. 1:**
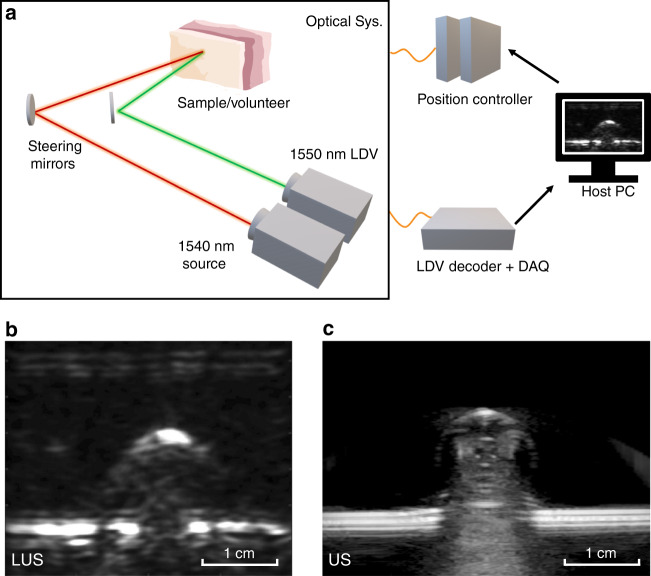
Simplified LUS system overview with LUS images on a gelatin phantom with a steel rod inclusion, with verification using a conventional clinical ultrasound imager. **a** Simplified schematic of the LUS system. Depending on the imaged subject, components may be placed on a translation stage. **b** Reconstructed LUS image of a gelatin phantom with a steel rod inclusion. **c** Conventional ultrasound image of the same gelatin phantom using a GE Logiq E9 system with a 9 MHz linear probe.

High-water-content gelatin phantoms with various inclusions were constructed to replicate the optical absorption characteristics of biological tissue in the infrared (IR) spectrum. Metallic spheres, rods, disks, and square-shaped inclusions were embedded in the phantoms to evaluate the LUS system imaging capabilities. The optical source and detection spots were mechanically calibrated, controlled, and collocated using steering mirrors and linear stages. Free-space control of the source and detection spots enables both 2D and 3D imaging. The time-series output of the LDV was recorded through a digital oscilloscope connected to the host computer. A simplified LUS system architecture is shown in Fig. 1a.

**Fig. 2:**
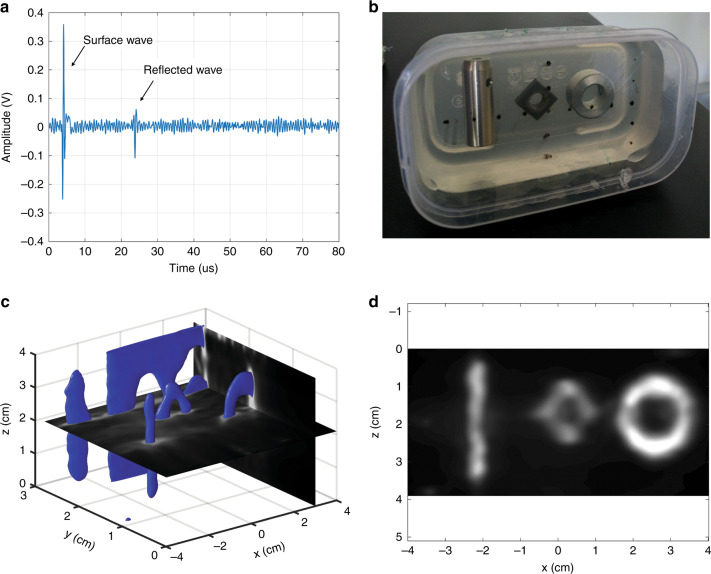
LUS image results demonstrating the 3D imaging capabilities of LUS over a 2D surface scan on a gelatin phantom with multiple inclusions. **a** Single LUS time trace on a gelatin phantom with metal inclusions. **b** Gelatin phantom used to evaluate 3D LUS imaging capabilities. **c** Reconstructed 3D LUS image of the 3D gelatin phantom with segmentation to emphasize the top surface of each inclusion. **d** Front XZ projection of the reconstructed 3D LUS volume.

As shown in Figs. 1b and 2c, the image reconstruction of LUS line scans and raster scans using a synthetic aperture focusing technique (SAFT) algorithm in conjunction with a coherence factor reconstructed both 2D and 3D images of the gelatin phantoms^37^. The LUS images were compared against a clinical GE Logiq E9 ultrasonic imager with a 9 MHz center frequency linear probe. By inspection, the LUS images and conventional B-mode images show broad feature agreements (Fig. 1b/c). However, the LUS system was also able to generate 3D volumetric images by raster scanning the LUS source and detector spots on the phantom surface (Fig. 2c). Prior LUS research imaged phantoms with embedded objects/tissue but relied on added retroreflective material on the phantom surface to enhance the optical reflectivity for the optical detector^39,42^.

### Ex vivo Animal Imaging

**Fig. 3:**
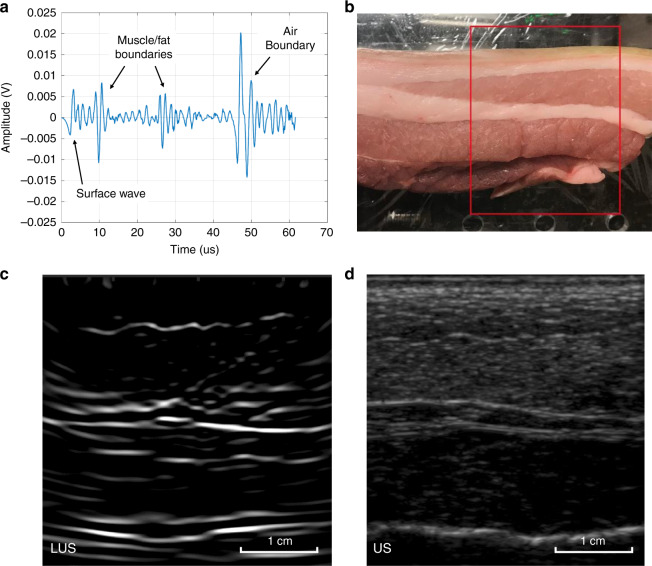
LUS image slice on porcine tissue to evaluate LUS performance on more optically representative tissue. Conventional ultrasound images verify features detected in LUS images. **a** Single LUS time trace on a porcine tissue sample, with multiple boundary echoes labeled. The initial surface wave echo was attenuated to improve the contrast of the other echoes. **b** Porcine tissue sample used for LUS imaging. The image region is outlined in red. **c** Reconstructed LUS image of the porcine tissue sample, showing multiple reflecting tissue layers. **d** Matching conventional ultrasound image of the porcine tissue, validating features seen in **c**.

Excised porcine abdominal tissue was imaged in the LUS system. Using porcine tissue as a human analog in biomedical research is well established, including for use in toxicology, immunology, wound healing, and radiation^47,48^. Porcine skin is similar to human skin in both anatomical structure and optical composition^49^. Porcine abdominal tissue was obtained from the local market without specialized preparation to include natural skin variations. For each sample, the epidermis, dermis, subcutaneous fat, and muscle layers were clearly visible. Similar to phantom imaging, LUS line scans were completed on each sample. Reconstructed LUS images with verification from conventional ultrasound are shown in Fig. 3c, d. No damage or marking was visible on any tissue surface after the LUS experiments.

The reconstructed LUS image confirms that LUS is sensitive to soft-tissue features at eye- and skin-safe optical exposure levels. In the LUS image (Fig. 3c), highly reflective air–tissue interfaces are visible at ~4.5 cm, while weakly reflective soft-tissue boundaries such as skin-fat and fat-muscle interfaces are also present. The subcutaneous fat layer and multiple muscle-fat boundaries are clearly present in the LUS image. Conventional ultrasound imaging (Fig. 3d) and visual inspection (Fig. 3b) verify all boundaries and features detected in the LUS images. Dominant soft-tissue boundaries are present in both the LUS and conventional ultrasound images at 1 cm, 2 cm, and 3.5 cm. In particular, the contour of the first reflecting muscle-fat layer at 1 cm matches in both the LUS and conventional images.

### In vivo human LUS imaging

**Fig. 4:**
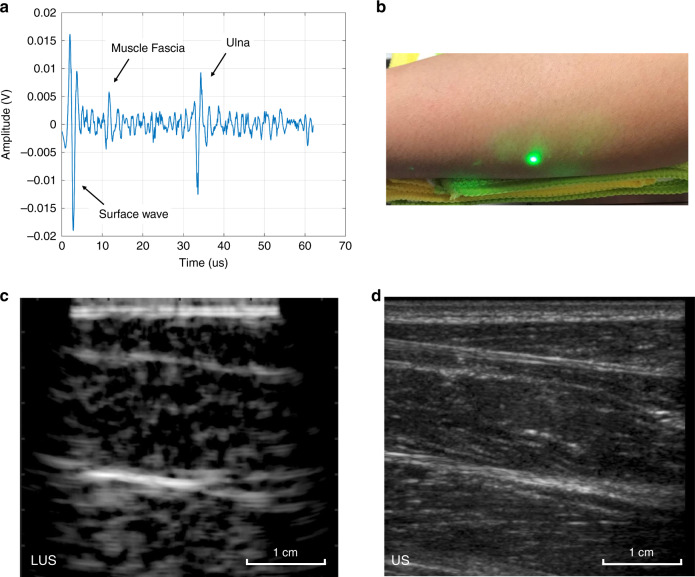
LUS images demonstrating LUS capabilities for in vivo human imaging at eye- and skin-safe optical exposure levels. Conventional ultrasound verifies soft- and hard-tissue features detected in LUS on the forearm. **a** Single time trace from LUS imaging of a volunteer’s forearm. **b** Photograph of a volunteer’s forearm region, imaged using LUS and conventional ultrasound. A green tracking laser from the LDV is also visible. **c** Reconstructed LUS image of a volunteer’s forearm with multiple visible tissue layers. **d** Matching conventional ultrasound image of the volunteer’s forearm using a GE Logiq E9 system and a 9 MHz linear probe confirming features detected in the LUS image.

The MIT Committee on the Use of Humans as Experimental Subjects (COUHES) approved the human LUS imaging protocol. Four volunteers’ forearms were imaged using the LUS system. Consent from each volunteer was obtained prior to LUS and conventional ultrasound imaging. Both the source and detection lasers were verified for safety against the ANSI standard. Similar to previous experiments, conventional ultrasound imaging using the GE Logiq E9 system followed each LUS imaging session for feature verification. LUS and conventional scans were completed on the inside and backside of volunteers’ forearms. No volunteer reported any sensation, discomfort, or tissue change during or following any LUS imaging session. Within the reconstructed LUS image shown in Fig. 4c, tissue features such as muscle fascia boundaries and the bone surface are clearly detected. In Fig. 4c, the muscle fascia boundary is present from 0.5 cm to 1 cm, and the surface of the ulna is present from 2 cm to 2.5 cm. Comparing LUS with conventional ultrasound, the same soft-tissue and bone features are present at the same depth, verifying that the LUS system can detect features presently detected by conventional ultrasound.

## Discussion

### LUS Results and Human Imaging

The LUS imaging results for porcine and human tissue demonstrate the capability of LUS to remotely and deeply image biological tissue at safe optical exposure levels. The tissue boundaries detected by LUS are in agreement with the tissue boundaries detected by conventional ultrasound. Components of LUS technology have been explored in prior research, but a full demonstration of LUS on a human subject has not previously been presented. The human LUS imaging presented here (Fig. 4c) is the first instance of LUS imaging on a human subject in vivo, and volumetric LUS imaging (Fig. 2c) demonstrates LUS capabilities for 3D imaging. By restricting optical penetration and selecting optical wavelengths that maximize both the generation and detection of acoustic waves, we can demonstrate LUS imaging on tissue-mimicking phantoms, excised porcine tissue, and human subjects without any surface enhancements for the optical source or detector performance. Validation of the LUS results on human subjects is a significant step toward proving the clinical viability of LUS and motivates further LUS research and development.

LUS imaging in porcine tissue (Fig. 3c) shows a clear and coherent boundary layer in the first 1.5 cm, but deeper layers present significant artifacts near the boundary. The same effect is visible in the human LUS image (Fig. 4c) for deeper layers. These depth-dependent artifacts are likely due to the lack of elevation focusing in LUS. While a conventional ultrasound probe is elevation focused to minimize out-of-plane reflections, optical sources and receivers are unfocused and become sensitive to more out-of-plane reflection at deeper imaging depths. More artifacts are visible in the human LUS image (Fig. 4c) than in the porcine LUS image (Fig. 3c) and can be attributed to patient motion and variations in optical backscatter from the skin. Overall, less acoustic speckle is present in the LUS images due to the lower imaging frequency, bandwidth, and lateral resolution of LUS than those of conventional ultrasound. Currently, LUS performance is limited by the acoustic frequency generated by the optical source and the detector sensitivity. The frequency of LUS sources is determined by the impinging optical wavelength and tissue absorption, with bandwidth limits due to the acoustic attenuation and tissue surface roughness; the expected source bandwidth for LUS at 1540 nm is ~1.5 MHz^45^. For optical detection, noncontact interferometric methods are limited by the optical backscatter from the tissue surface. Optical detectors have more than sufficient bandwidth for ultrasound imaging but are generally reliant on ideal stationary reflective surfaces for measurement, without consideration of human safety^35^. Based on our human subject experiments, there is significant variation in the optical backscatter, as the detector was used to scan the skin tissue. Optical reflectance on human subjects has been reported for optical wavelengths between 250 and 2500 nm, but the measurement of backscatter specularity is lacking^50,51^. Full skin reflection characteristics must be investigated to design specialized optical detectors for clinical LUS. For the LUS system reported here, the 1550 nm LDV was specifically selected to maximize permissible optical backscatter from the skin while maintaining safety. Adaptive focusing could further reduce variability associated with skin variations and patient motion, but feedback control of the optical output based on the optical backscatter will be necessary.

At this point, the LUS images presented here are comparable to images presented at the incipient stages of medical ultrasound imaging decades ago^1^. Quantitative image comparison of the current LUS technology against modern medical ultrasound is still premature. The conventional ultrasound images presented here use a 9 MHz center frequency transducer with multielement beamforming and are of expectedly higher image quality and resolution than those of the LUS images. Nevertheless, similar structures and dimensions are consistently observed in both modalities. While further work remains prior to commercialization and clinical use, the core enabling technologies of LUS are available. Discussed in later sections, recent research advances in laser technology, silicon photonics, and hydrogels could accelerate future LUS development.

### Optical system design

From prior literature, LUS sources can be approximated by an equivalent disk transducer below the tissue surface, with the geometry determined by the parameters of the impinging light and absorption characteristics of the irradiated tissue^22,23^. With proper thermal and mechanical stress confinement, the equivalent disk is prescribed, in diameter, by the impinging optical beam diameter, and in thickness, by the optical penetration depth (inverse of the tissue optical absorption coefficient at the impinging optical wavelength)^22^. Thus, the central frequency of the converted acoustic wave and the conversion efficiency are directly determined by the optical absorption coefficient^22^. For practical applications, the beam diameter should be larger than the penetration depth such that the dominant propagation direction of the converted acoustic wave is directed into the tissue. For maximum conversion efficiency, the optical absorption coefficient should equal the acoustic wavenumber of the desired acoustic frequency to be generated^22^. For biological tissue, the stress confinement conditions for LUS are satisfied by nanosecond pulsed infrared sources, restricting optical conversion at the tissue surface using high optical absorption regions of water^45^. To generate acoustic frequencies relevant for clinical ultrasound (1–10 MHz), ideal optical wavelengths for LUS source generation are ~1500 and 2000 nm, corresponding to peaks in the water absorption spectrum. At 1500 nm, the optical absorption water is ~40 cm^−1^, which corresponds to a converted acoustic source near 1 MHz. Prior work reports that both 1500 nm and 2000 nm pulsed sources generate acoustic waves with bandwidths up to 1.5 MHz^45^. However, the MPE limit for nanosecond sources at 2000 nm is an order of magnitude lower than that at 1500 nm, 0.1 J/cm^2^ vs. 1 J/cm^2^, respectively^46^. Thus, the 1540 nm optical source was selected to maximize the converted acoustic source amplitude while remaining within the safety limits. Following the disk transducer model, the optical beam diameter dictates the beam profile of the converted acoustic source. For the 2 mm diameter optical source on tissue with an expected frequency of ~1.5 MHz, the acoustic beam width is approximately 60 degrees. A narrower beam spread can be generated using larger optical beam diameters, but significantly higher optical energy is required to maintain the overall fluence.

For optical detection, optical backscatter from the tissue surface must be maximized. Prior measurements of optical reflectance from human skin report that wavelengths between 500 nm and 1200 nm offer the highest optical reflection factor^46,50,51^. However, the safety MPE limits must be considered again. For a 100 µs measurement duration (corresponding to an imaging depth of 7.5 cm in tissue), assuming similar photodetector responsivity across the spectrum, if a tissue surface is irradiated at the maximum MPE limits for every spectral region, the maximum quantity of reflected light to a detector is still in the far infrared, between 1500 and 1800 nm. Thus, the selected 1550 nm LDV balances detection sensitivity with subject safety. Considering subject safety and acoustic performance, ~1500 nm is the optimal spectral region for both LUS transmission and detection and is able to leverage the commercial abundance of 1550 nm optical components from the telecommunication industry^45^.

LUS performance is directly linked to the intrinsic tissue properties of human skin. The absorption and reflection parameters dictate LUS transmission and detection, respectively. The tissue surface roughness limits the LUS transmit bandwidth and may contribute to variation and specularity in optical reflection, which degrades the optical detection sensitivity. Skin tissue inhomogeneity from pigmentation and melanin may further affect the LUS performance and warrant further investigation. Averaging multiple traces improves the overall signal-to-noise ratio, but the method is limited by MPE limits and increases the overall imaging time; poor or a lack of optical backscatter from the tissue surface cannot be resolved by averaging. Surface treatment, adaptive focusing, or multichannel optics may be necessary for clinical LUS systems to mitigate tissue variability. Furthermore, unlike fixed-geometry ultrasound arrays, the equivalent optical LUS array conforms to the local tissue geometry. The array geometry for the LUS images presented can be approximated as planar, but the imaging of large tissue regions with significant local curvature requires active localization of the sources and detectors to accurately reconstruct an image.

### Enabling LUS technologies

Future LUS development should focus on component improvements as well as surface treatments to enhance multiple facets of LUS. An amplitude-modulated optical source can excite higher acoustic bands to improve the image resolution^22^. Implementing a fast amplitude-modulated optical source could improve the LUS imaging depth, bandwidth, and resolution; in addition, conventional ultrasound techniques used to amplify the acoustic signal-to-noise ratio, such as pulse compression, matched filtering, and source encoding, can be leveraged. Currently, both LUS and PA are limited by the existing laser technology^16–18,41,52^. Beyond amplitude modulation, the parallelization of optical sources and receivers to enable optical transmit and receive beamforming techniques will be a critical turning point, similar to how piezoelectric arrays enabled and revolutionized clinical ultrasound imaging. Since the spatial locations of LUS sources are restricted to the tissue surface and can be known *a priori*, transmit beamforming is possible only in LUS and not in penetrating PA. The adaptation of computer vision techniques such as 3D imaging and tracking technologies could make large-volume LUS imaging and optical beamforming feasible. Optical spot tracking is also required if patient motion is significant during data acquisition. With sufficiently fast data acquisition and coverage, LUS systems could behave similar to a body volume camera, capable of simultaneous 3D imaging of both the external and internal tissue geometries. The silicon photonics industry, fueled by sensors required for autonomous car operations, has developed chip-scale, solid-state, steerable laser technology applicable for both PA and LUS imaging^53–57^. Since the ideal operating range for LUS is near 1500 nm, communication and silicon photonic innovations may directly impact LUS system development.

As discussed previously, the LUS system performance is subject to the optical and acoustic properties of the tissue. A minor surface treatment layer could bypass the tissue limitations. Radio frequency (RF) coils and contrast agents are commonly used for MRI and CT, respectively; designing a surface treatment layer for LUS is not inconceivable. Enhanced optical reflection is commonly achieved with retroreflective dust or tapes^39,42^. However, retroreflective dust is a respiratory irritant, and tape or dust on the tissue surface impedes the LUS source. Gel or gel pads embedded with retroreflectors could be designed to enhance optical detection without interference to the optical source; the bulk water content of the gel can preserve the LUS source characteristics in the IR spectrum, while embedded reflectors can enhance the optical backscatter. Furthermore, source bandwidth limitations due to the tissue roughness can be bypassed by generating the LUS source in the gel layer. Gel or gel pads routinely used for ultrasound imaging can be augmented for LUS imaging. Flexible hydrogels could also be designed for LUS; the bulk water content of hydrogels mixed with retroreflective particles can conform to the tissue surface and enhance the LUS performance^58^. The treatment layer could expand the LUS source bandwidth, improve the optical detection sensitivity, and permit higher optical exposure limits. However, irradiance exceeding the MPE limits on the treatment layer may mandate additional safety measures such as enclosure or eye protection. New optical designs should be evaluated to accommodate the use of surface treatments in LUS imaging.

### Outlook

Based on these encouraging results, LUS inspires confidence for further research and development. Human LUS images verify the feasibility of LUS for in vivo anatomical imaging without compromising patient safety. LUS is sensitive to both hard and soft anatomical features, similar to conventional ultrasound, but is fully noncontact. The real-time remote sensing of biological tissue would find broad applicability in nonintrusive patient monitoring, contact sensitive imaging (elastography and musculoskeletal disease tracking), and intraoperative applications. Current embodiments of LUS for research are generally single-point transmission and detection due to the high cost and complexity. Clinical iterations of LUS will require multipoint optical transmission and detection to amplify the acoustic source amplitudes and reduce the data acquisition time. Analogous challenges existed in the nascent stages of conventional ultrasound imaging. Scaling from mechanically scanned single-element systems to highly parallelized real-time clinical imagers took decades of research and development^1^. A similar pathway is likely for LUS – parallelization of single- to multipoint laser technology. The initial human results are encouraging; rapid advances in related industrial sectors will establish the technologies necessary to enable clinical implementation of LUS.

## Materials and methods

### System configuration and imaging

A 1540 nm passively Q-switched pulsed laser (Optitask OT-37) was selected for LUS source generation. A commercially available class-2 1550 nm laser Doppler Vibrometer (Polytec RSV-150) with a green tracking laser (<1 mW) was selected for the optical detection of ultrasonic signals. The bandwidth of the LDV is 2.5 MHz, with 49 mm/s/V sensitivity. The minimum focused diameter for the LDV is 135 µm. Both lasers are within the respective pulsed and continuous MPE limits in the IR spectrum for eye and skin safety. A significant margin of safety is available to engineer custom LUS solutions with higher power and faster repetition rates but is outside the scope of the human LUS feasibility study presented here.

For each slice or volumetric image, the optical source and detection points were collocated and sequentially scanned on the sample surface linearly or rastered, respectively. Optical steering and collocation were completed using fast steering mirrors (Optics in Motion LLC, OIM102.3) and linear stages (Sigma Koki Co, SGSP26–200 stage, SHOT-702 motion controller). The spatial sampling frequency in each instance satisfies the Nyquist limits for the expected 1.5 MHz maximum acoustic frequency to prevent grating lobe interference. A scan length of ~5 cm at 0.5 mm pitch was completed for each imaging instance. The aperture length for LUS was the same as that for the conventional ultrasound probe used for verification. For volumetric LUS imaging, a phantom was scanned with a 4 cm by 8 cm mesh grid with a 0.5 mm pitch. For each sampling point, 50 time traces in total were recorded and compiled for postprocessing and image reconstruction. Data acquisition from the LDV was recorded on a digital oscilloscope (National Instruments, NI PXIe-5170R, 14-bits, 250 MHz max bandwidth) nested in an NI PXIe-1073 chassis. Post data acquisition, an SAFT algorithm with a coherence factor – to minimize the side-lobe artifacts – was used to reconstruct images for each LUS dataset^37^.

### Phantom composition

The LUS phantoms consist of porcine gelatin (6% by weight, Sigma-Aldrich, gel strength 300, Type A) dissolved in deionized water at 85 °C. To match typical tissue sound speeds, 1-propanol (2% by weight, Alfa Aesar, A19902) was dissolved in the gelatin mixture. The solution cooled to 45 °C prior to pouring into molds with the desired inclusions. The solution was degassed in the molds and left to solidify at room temperature. All phantoms were sealed and stored at 4 °C and used in the experiments within one week of construction to avoid sound speed changes due to water loss over time.

### Animal sample preparation

The use of animal tissue was approved by the MIT Committee on Animal Care, protocol number E17–09–0320. Porcine abdominal tissue was obtained from the local market. The sample tissue strips include skin and multiple layers of visible muscle and fat without deep cartilage or bone inclusions. The skin surfaces were not specially selected or treated to include natural skin variations in the experiments. The strips were ~5 cm thick and cut to 25 cm lengths prior to LUS imaging.

### Human LUS

All human LUS experiments were completed with approval from the MIT Committee on the Use of Humans as Experimental Subjects (protocol # 1702850719R001). As an extra precaution, all laser component optical outputs were measured using an optical power meter (Ophir Nova II). As dictated by the Institute Review Board (IRB), all optical outputs were independently verified to be eye and skin safe. Laser safety glasses were still available if requested by any volunteer. All personal identifiers were removed from the LUS and conventional US data to preserve volunteer anonymity. Each imaging instance covered an ~5 cm line across a volunteer’s forearm, with conventional ultrasound imaging completed at the same location immediately following LUS imaging. The forearm was selected for ease of accessibility by the LUS system and significant collections of soft- and hard-tissue features.

### Conventional ultrasound validation

A standard clinical system, GE Logiq E9, with a 9 MHz linear probe provided verification of the LUS images using conventional ultrasound methods. The acquisition process with the conventional system consisted of applying ultrasound gel (Aquasonics 100) to the probe and acquiring B-mode ultrasound images at the same location as that of the LUS scan. Although the conventional ultrasound system operates at significantly higher frequencies and leverages beamforming, gross comparisons of large soft-/hard-tissue features, such as fascia, arteries, muscles, tendons, and bones, are still possible between LUS and conventional ultrasound. For this article, B-mode ultrasound serves as the gold standard to verify the tissue features detected using LUS.

## Data Availability

The datasets generated and analyzed in the article are available from the corresponding author upon reasonable request.
